# Placental protein-1 (plac1) modulates immune tolerance in mammary tumor cells

**DOI:** 10.1186/2051-1426-3-S2-P221

**Published:** 2015-11-04

**Authors:** Hongyan Yuan, Robert Glazer, Xiaoyi Wang, Lu Jin, James Li, Nairuthya Vijayendra, Venkata Doodala, Spencer Weiss

**Affiliations:** 1 Georgetown University, Washington, DC, USA; 2 Thomas Jefferson High School for Science & Technology, Alexandria, VA, USA; 3 Thomas Jefferson High School for Science & Technology, Great Falls, VA, USA

## 

Plac1 is an X-linked (Xq26) trophoblast gene expressed at high levels in the placenta, at low levels in the testis, but not in other normal somatic tissues. However, it is re-expressed in several malignancies, including breast, colon, lung, gastric, liver and endometrial cancers as well as in most human cancer cell lines. Plac1 contains HLA-A2-restricted epitopes capable of eliciting a cytotoxic T lymphocyte (CTL) response against human breast cancer cells, and colorectal cancer patients with a Plac1-specific CTL response demonstrate long-term survival. To explore the role of Plac1 in cancer, mouse mammary tumor E0771 cells expressing high levels of Plac1 were transduced with a lentivirus expressing a Plac1 shRNA (E0771/shPlac1). E0771/shPlac1 cells exhibited >98% reduction in Plac1 mRNA, which resulted in 50% inhibition of cell growth as measured by cell count or colony formation. Isografts of E0771/shPlac1 cells in syngeneic C57BL/6 mice did not result in tumor formation, but grew similar to control cells in SCID mice, suggesting a role for Plac1 in immune tolerance. This was confirmed by gene expression profiling, which revealed that Plac1 upregulated inflammatory chemokines, including Cxcl1, Ccl7, Ccl2, Cxcl10, as well as Ly6g and PD-L1, markers of myeloid-derived suppressor cells (MDSC) and regulatory T cells (Treg), respectively. E0771 tumors in B6 mice exhibited high expression of Plac1 and PD-L1 by IHC, and treatment of tumor-bearing mice with a PD-L1 mAb inhibited tumor growth. To assess the role of Cxcl1, a driver of MDSC activation through its receptor Cxcr2, tumor-bearing mice were treated daily with 2 mg/kg of the Cxcr2 antagonist, SB225002, which resulted in 50% inhibition of tumor growth. These results are the first to suggest that Plac1 increases immune tolerance to tumor growth by sustaining the activation of MDSC and Treg, in part through increased Cxcl1 and PD-L1 expression. These data also suggest that Plac1 may temper autoimmunity in the developing fetus in part by regulating MDSC and Treg activity.

